# Antifungal Activity of Phenolic and Polyphenolic Compounds from Different Matrices of *Vitis vinifera* L. against Human Pathogens

**DOI:** 10.3390/molecules25163748

**Published:** 2020-08-17

**Authors:** Giovanna Simonetti, Elisa Brasili, Gabriella Pasqua

**Affiliations:** Department of Environmental Biology, Sapienza University of Rome, P. Aldo Moro 5, 00185 Rome, Italy; giovanna.simometti@unrioma1.it (G.S.); elisa.brasili@uniroma1.it (E.B.)

**Keywords:** human fungal pathogens, *Vitis vinifera*, berry skins and seeds, leaves and canes, not-fermented and fermented-pomaces, procyanidins, phenolic compounds

## Abstract

Phenolic compounds, the most widely distributed class of natural products in the plants, show several biological properties including antifungal activity. Phenolics contained in grapes can be classified in two main groups, flavonoids and non-flavonoids compounds. Variability and yield extraction of phenolic and polyphenolic compounds from different matrices of *Vitis vinifera* depends of cultivar, climate, soil condition and process technology. Unripe grapes, berry skins and seeds, leaves, canes and stems and not-fermented and fermented pomaces represent large reusable and valuable wastes from agricultural and agro-industrial processes. This review summarizes studies that examine the extraction method, chemical characterization, and antifungal activity of phenolic and polyphenolic compounds from edible and non-edible *V. vinifera* matrices against human fungal pathogens. In the world, around one billion people have fungal diseases related to skin, nail or hair and around 150 million have systemic diseases caused by fungi. Few studies on antifungal activity of plant extracts have been performed. This review provides useful information for the application of *V. vinifera* phenolics in the field of antifungals for human use.

## 1. Introduction

Every year fungi infect billions of people. Despite the high number of infections and high mortality rates, fungal diseases remain poorly studied. Traditional antifungals used to treat fungal diseases are not really effective, and very few new antifungals are currently in development. The available drugs against fungal diseases are few and increased resistance to these drugs has led to uncertainties about the efficiency of treatment in the future [1]. On the other hand, the pharmaceutical industry has disregarded investments in new and effective antifungal agents. The development of new antifungals is necessary to fight fungal diseases efficiently. In this context, the interest in alternative therapeutic interventions has been growing and the use of plant extracts as adjuvants in antimicrobial therapy has been highlighted in recent years [2]. There are few studies on antifungal activity of plant extracts as compared with bacterial infectious diseases [3]. Most of the studies are performed against plant fungal pathogens and against food fungal contaminants [4,5,6]. To date, only a few extracts or compounds of plant origin have been shown to have antifungal activity against human and animal pathogens [7,8,9,10,11,12,13]. Regarding the antifungal activity of phenolic and polyphenolic compounds, the most studied are obtained from different matrices of *Vitis vinifera* L.

The grapevine (*Vitis vinifera* L.) is one of the most cultivated fruit crops in the world based on hectares cultivated and economic value, including around 23,000 cultivars. Grapes are used not only for wine but also for fresh and dried fruit, as well as for juice production. In the vine-wine industry, agro-waste is generated in large quantities. The wastes are of almost zero value and in some cases require an additional cost for disposal. All these wastes, containing phenolic compounds, are a source of added-value products. Some *V. vinifera* extracts from different matrices have shown antifungal and antibacterial properties, corroborating the traditional therapeutic uses of this plant. Wines and winery byproducts and their bioactive components are active against human pathogenic bacteria, viruses, fungi in relation to their composition [8]. As indicated by Fraternale et al. [9] ethanolic extracts obtained from *V. vinifera* L. tendrils showed in vitro antifungal activity against plant pathogenic fungi such as *Fusarium* species with minimum inhibitory concentration (MIC) values ranging from 250 to 300 ppm, while the basidiomycete fungus *Rhizoctonia solani* was the most resistant, with a MIC value of 500 ppm. There are several studies published on the antifungal activity against human pathogens, most of them on *Candida,* of *V. vinifera* extracts from grape wastes and byproducts derived from agricultural and agro-industrial processes. In the following sections chemical characterization of crude extracts rich in phenols and polyphenols from different matrices of *V. vinifera* and their antifungal activities will be examined and discussed. A literature search was performed using Google Scholar and PubMed, and by finding the keywords “flavonoids”, “antifungal agents”, “procyanidins”, “flavonols” or “flavones” in “Title/Abstract/Keywords”, with a date cut off, and checking case–control studies, placebo, clinical, in vitro and in vivo studies that examined the relationship between phenolic compounds and their antifungal effects. Each antifungal mechanism was considered and arranged in an appropriate section in the review. The present review is also a critical literature revision of extraction methods of phenolic compounds from different grape matrices, and their chemical composition, with emphasis of their antifungal activity.

## 2. Chemical Characterization of Phenols and Polyphenols in Different Matrices of *V. vinifera* L.: Berry Skins, Seeds, Leaves, Stems, Canes and Not-Fermented and Fermented Pomaces

The phytochemical composition of a grapevine includes a great variety of flavonoid and non-flavonoid compounds. Grape flavonoids include flavonols, flavones, flavan-3-ols, flavanones and anthocyanidins. Flavonoids consist of a skeleton of 15 carbon atoms comprising of two aromatic rings bound through a three-carbon chain. The non-flavonoids comprise of stilbenes and phenolic acids. The latter group includes hydroxybenzoic acids and hydroxycinnamic acids, containing seven and nine carbon atoms, respectively. Phenolic compounds have been found in several parts of grape berries such as skins, pulp and seeds and it was observed that their content depends on climatic and geographical factors, as well as stages of ripeness or cultural practices [14]. Grape variety is also a key parameter of the berry phenolic composition [15,16,17]. The most abundant phenolic compounds in grape skins are flavonols, while grape seeds are rich in flavan-3-ols [18,19]. Anthocyanins found in the skin are directly responsible for the color of red grapes cultivars. In the seeds, flavan-3-ols (monomeric catechins and proanthocyanidins) are the main polyphenolic compounds [20,21,22]. Grape seed extract (GSE) is recognized as a complex mixture of monomeric, oligomeric and polymeric flavan-3-ols. The principal monomers identified are (+)-catechin, (−)-epicatechin, (−)-epicatechin gallate, (−)-epigallocatechin and (−)-epigallocatechin gallate. Oligomeric proanthocyanidins consist of two or more monomers, chemically bonded. The dimeric procyanidins are often referred to the B-series. Different dimers (B1–B8), dimers esterified with gallic acid (B1-3-O-gallate, B2-3-O-gallate) and trimers (C1, C2) were identified in grape seeds [23]. The content of flavan-3-ols in seed grapes is influenced by several factors mainly cultivar, irrigation, soil fertilization, delayed harvest and storage conditions [16,24]. Giannini et al. [17] studied seed extracts in terms of total phenols, monomeric, dimer, polymeric and gallate esters flavan-3-ols composition from 15 red and white grapevine cultivars/clones. It was demonstrated that the amount of phenols accumulated in white grape seeds was higher than the amount in red grape seeds. The polymeric procyanidins were significantly higher in 2014 in all the cultivars, while the gallate esters were significantly higher in 2013. These differences were attributed to different climatic conditions in the years [17].

GSE has become popular in recent years as a nutritional supplement for its potentially beneficial effects for human health [20,22,23,24,25,26,27,28]. Very low studies for non-edible organs of *V. vinifera,* such as leaves and canes, which represent a large reusable and valuable waste from pruning, have been conducted. For this reason, numerous attempts for isolation, identification and quantification of phenolic compounds from a grapevine have been ongoing. In a recent review, the chemical diversity of polyphenols within *V. vinifera* vegetative organs was provided [29]. The review includes an overview of compounds identified in the canes, stems and leaves and an estimation of their levels. At least 183 phenolic compounds have been identified, in the canes, stems and leaves, including stilbenes (from monomers to hexamer), hydroxycinnamic acids, hydroxybenzoic acids, flavan-3-ols, anthocyanins, flavanones, flavonols, flavones and coumarins. As the main flavonoid compounds, leaves contain flavonols for 83% and stems/canes flavan-3-ols for 62%. The main flavonols and hydroxycinnamic acids in the leaves are quercetin-3-O-glucuronide, quercetin-3-O-galactoside, quercetin-3-O-glucoside and caftaric acid; catechin, epicatechin, procyanidin B1 and quercetin-3-O-galactoside are the most represented flavan-3-ols and flavonols in the stems and canes. The main stilbenes as trans-ε-viniferin, trans-resveratrol, isohopeaphenol/hopeaphenol, vitisin B and ampelopsins have been found primarily in the roots, the canes and the stems, whereas the leaves present a low concentration of these compounds [30,31]. Unripe grapes are a large source of waste material obtained from the wine industry. Unripe grapes are discarded during cluster thinning used to increase the size of table grapes and improve berry quality [32]. Recently, Simonetti et al. [33] showed that three main classes of phenolic compounds, as flavan-3-ols, cinnamoyl derivatives and flavonols, are present in green grapes. Flavan-3-ols, which ranged from 3.3 to 6.8 mg/g fresh weight, consisted of monomers (+) catechin and (−) epicatechin, the dimer procyanidin B2 and a more polar isobar, several mono- and di galloylated dimers, a non-galloylated trimer and two groups of polymers. The concentration of flavonols and cinnamoyl acids was from one to three times lower than the amount of flavan-3-ols. Concerning the flavonols, the quercetin-3-O-glucuronide was the predominant compound in all the samples. Grape pomace is another abundant waste from the wine industry, consisting of skin, seeds and stalks and representing around 25% of the total grape weight utilized in the winemaking process. In Italy, France and Spain, where wine production is more relevant, the annual grape pomace generation can reach nearly 1200 tonnes per year. It is important to make a distinction between not fermented and fermented pomaces. The white vinification, without maceration step of the grape skins in the must, produces not fermented pomace, while the red vinification process with the grape skin maceration step in the must leads to fermented pomace. Not-fermented grape pomaces are characterized by a high content of phenolics with respect of fermented pomaces, due to an incomplete extraction during the winemaking process [34].

It has been reported that after processing, approximately 70% of the phenolic content is preserved in the grape pomace [35,36,37]. Both seedless pomace (residual pulp, skin and stem) and the seeds are rich in bioactive compounds. The most abundant phenolic compounds in wine pomace are represented by anthocyanins concentrated mainly in the skin, and flavonols mainly present in the seeds (56–65% total flavonol) [38]. Several non-flavonoid phenolic acids compounds were identified in wine pomace including protocatechuic acid found in grape skins and only in red grapes, and tartaric esters of caffeic, coumaric and ferulic hydroxycinnamic acids. In the skin of white grapes only the trans isomer of hydroxycinnamic acid was detected and in small quantities.

## 3. Extraction of Phenolic and Polyphenolic Compounds from *V. vinifera* and Detection Methods

The extraction procedure is the primary determinant for the separation and recovery of phenolic and polyphenolic compounds with antifungal activity from *V. vinifera.*

The effectiveness of extraction process depends mostly on the type matrix and the chemical properties of the phenolics and polyphenolics, including molecular structure, polarity, concentration, number of aromatic rings and hydroxyl groups. The diversity in the chemistry of phenolics in a sample is also related to the concentration of simple and particle size that strongly affect the different proportions of phenolic acids, flavonoids, anthocyanins and proanthocyanins to be extracted. For this reason, it is difficult to choose a single method of extraction, separation and purification of phenolic and polyphenolic compounds.

It is known that the extraction of phenolic compounds is dependent upon two steps, the dissolution of each chemical compound at the cellular level in the plant material matrix, and their diffusion in the external solvent medium. The type of solvent plays thus a major role in the extraction process and several reports on the optimization of the yield of phenolic extracts using different solvents and extraction methods were published. To obtain different total phenolic values, it is useful to adopt different solvent mixtures in the extraction process, due to their differing efficacies in the solubilization of phenolic compounds and penetration of the sample matrices [36,39,40,41]. Procyanidins from *V. vinifera* represent the group of polymerized flavan-3-ols with the best antifungal activity. In Table 1, we summarized the *V. vinifera* matrices with the highest procyanidin content and total flavan-3-ols, extraction methods, solvent and yield. 

Generally, the most of extractions are performed with organic solvents and the most promising solvent for most of the flavan-3-ols seems to be 60–80% ethanol in water. However, at the present time, there is no reported grape extraction procedure utilizing ethanol as the unique solvent. The extraction time and temperature have also an important role improving the yield or shortening the extraction process. Analytical techniques commonly used in the determination of phenolic compounds are high-performance liquid chromatography (HPLC) in combination with ultraviolet detection (UV), electrochemical detection or mass spectrometry detection (MS). The detection limits can be achieved by using appropriate sample preparation and considering that the type of the matrix strongly affect the quality and quantity of results.

As demonstrated by Simonetti et al. [16], Italian GSE obtained from *V. vinifera* L. cv. Michele Palieri grown in conditions of reduced irrigation, extracted three times (24 h for each extraction) by the mixture EtOH/H2O (7:3 *v*/*v*) acidified with formic acid to pH 3 and finally dried (*t* ≤ 30 °C) showed a content of polymeric flavan-3-ols of 820 mg/g DW. Some studies show that a 50% aqueous solvent is the optimum concentration for the extraction of phenolic compounds, as well as being cost effective [42,43]. Due to the water solubility of many polyphenols, hot water could serve as an extraction solvent. Vergara-Salinas et al. [44] demonstrated that pressurized hot water at the laboratory scale was able to extract grape pomace before and after fermentation. Fermented pomace yielded more tannins than unfermented pomace but fewer anthocyanins. Relevant proanthocyanidin amounts were extracted only at 50 and 100 °C. Using higher temperatures and longer extraction times resulted in a sharp decrease of the polyphenol extraction yield.

Nawaz et al. reported that a double stage extraction utilizing 50% ethanol and 95% ethanol, added to an ultrafiltration step with 0.22 µm membrane pore size were the optimal conditions to extract the most of polyphenols from grape seeds [45]. Extraction at room temperature [46,47] and a shorter extraction time of less than 1 h [42,48] are most commonly used for phenolic extractions from wine residue. According to Bucic-Kojic et al. [48], 80–90% of total polyphenols collected within 200 min was extracted in the first 40 min of the extraction process. Cheng et al. [39] analyzed dried grape seeds, skin and pomace obtained from *V. vinifera* Pinot noir and Pinot Meunier grown in the cool climate of New Zealand. To obtain defatted samples, grape seeds and pomace were extracted with 100 mL of hexane for 1 h at room temperature (20 °C). The samples were then filtered, and the residues were air-dried to allow for the evaporation of hexane. The extraction process was carried out at 25 °C for 40 min using either acetone/water, ethanol/water or methanol/water at 1:1, *v*/*v* ratio in the dark with approximately 50 mL solvent at a constant stirring rate of 200 rpm. Liquid chromatography mass spectrometry (LC–MS) was used to analyze the extracts. Phenolic compounds were detected by a photodiode array (PDA) detector using wavelengths between 25 and 700 nm and quantifications were carried out at 520 nm. The analysis was monitored by electrospray ionization (ESI) interface. The extraction yield varied from 4.3% to 65.9% (*w*/*w*) depending on the extraction solvent as well as grape variety. The higher levels of catechins and their derivatives were obtained using methanol/water (50%). A series of glucoside derivatives from anthocyanins including delphinidin-3-glucoside, cyanidin 3-glucoside, petunidin-3-glucoside, peonidin-3-glucoside and malvidin-3-glucoside were also identified by LC–MS analysis. The total anthocyanins detected were expressed in malvidin equivalent (ME) ranged from 2.6 to 17.4 mg ME/g extract [39]. The possibility to obtain pure extracts has made the supercritical fluid (SC-CO_2_) extraction a very important tool in the research field. It is well-known that the quantity of the extract yield from supercritical CO_2_ extraction is affected by several operating parameters such as the CO_2_ pressure, CO_2_ mass flow rate, time of extraction and average particle size. Oliveira et al. [49] analyzed the chemical profile of Merlot and Syrah grape pomace extracts obtained by supercritical CO_2_ (SC-CO_2_) and CO_2_ added with a co-solvent at pressures up to 300 bar and temperatures of 50 and 60 °C. The main chemical compounds of extracts, identified by HPLC, were gallic acid, p-OH-benzoic acid, vanillic acid and epicatechin. Despite lower extraction yield results, the supercritical fluid extracts presented the highest antimicrobial effectiveness compared to the other grape pomace extracts due to the presence of antimicrobial active compounds. In several studies performed to analyze antifungal activity of grape seed extracts against *Candida* species, no detailed chemical analysis has been conducted to identify phenolic compounds [50,51].

## 4. Antifungal Activity of Crude Extracts, Phenolic and Polyphenolic Compounds from Leaves, Stems, Canes of *V. vinifera* Against Human Pathogenic Fungi

As described in Section 2 the main phenolic compounds in the leaves are flavonols for 83% and in the stems/canes flavan-3-ols for 62%. Stilbenes accumulate primarily in the canes and stems. An inducible antifungal compound in grapevine leaves is the pterostilbene (PTB); it is one of the most active antifungal compounds, even more than resveratrol and viniferins [52]. The fungal genus *Candida* is the most important human fungal pathogen. Candidiasis is the leading cause of morbidity and mortality worldwide and is the main systemic fungal disease. Candidiasis comprises of several categories of diseases, systemic or invasive diseases and muco-cutaneous infections such as intertrigo and vaginitis. Among *Candida* species, *C. albicans* is the most common species that causes human diseases. *C. albicans* infections are frequently associated with biofilm formation. Cells within the biofilms are much more recalcitrant to the conventional antifungal treatment than planktonic cells [53]. Antifungal activity of pterostilbene entrapped in poly(lactic-co-glycolic) acid nanoparticles (PLGA NPs) with diameters of 50 and 150 nm has been tested on the *C. albicans* biofilm. It has been observed that pterostilbene entrapped in PLGA NPs exerted a significantly higher anti-biofilm activity compared to their free forms. At 16 µg/mL, pterostilbene loaded in PLGA NPs reduced biofilm formation of 63% and reduced mature biofilm of 50% [54]. As for resveratrol, anti-*Candida* activity has been demonstrated at the concentration of 10–20 μg/mL by Jung et al. [55]. Moreover, resveratrol exhibited a fungicidal effect on *C. albicans* confirmed by scanning electron microscopy [55]. Resveratrol also showed inhibition of 80% growth of dermatophytes in particular against *Trichophyton mentagrophytes*, *Trichophyton tonsurans*, *Trichophyton rubrum*, *Epidermophyton floccosum* and *Microsporum gypseum* at a concentration of 100 μg/mL [56]. Resveratrol was potent to the same extent as amphotericin B against *Saccharomyces cerevisiae* KCTC 7296 with MIC value of 10–20 μg/mL and *Trichosporon beigelii* KCTC 7707 with an MIC value of 10 μg/mL, [56,57]. Dermatophytes including the genera *Trichophyton*, *Microsporum* and *Epidermophyton*, cause dermatophytosis worldwide. Dermatophytosis is the most frequent type of superficial mycosis in humans, attacking skin and nails and sometimes is extensive and difficult to treat [58].

*S. cerevisiae* is part of the normal flora as well as agent of invasive fungal infection. Ventoulis et al. [59] have reported that critically ill patients, to severe COVID-19, after *Saccharomyces* supplementation developed a *S. cerevisiae* bloodstream infection. *T. beigelii* is the etiological agent of white piedra, a superficial fungal infection of the hair shaft [60].

Houille et al. [61] evaluated anti *Candida* activity of resveratrol oligomers, viniferins, purified from *V. vinifera* canes and several analogues obtained through semi synthesis using methylation and acetylation. Neither the natural oligomers such as trans-ε-viniferin, cis/trans-vitisin B, ampelopsin A and hopeaphenol, nor the derivatives of trans-ε-viniferin were active against *C. albicans*. However, the dimethoxy resveratrol derivatives exhibited antifungal activity against *C. albicans* with MIC values of 29–37 μg/mL and against 11 other *Candida* species. It is to be emphasized, however, that resveratrol is not effective in vitro against *Candida dubliniensis, Candida glabrata, Candida tropicalis, Candida parapsilosis* and *Candida krusei* [61,62].

*Aspergillus* species are etiological agents of wide spectrum of infections in humans, mainly in immunocompromised individuals [63].

Methanolic crude extract from leaves *V. vinifera* was evaluated against *Aspergillus niger.* Results showed MIC values of 75.4 µg/mL against *A. niger* [64]. Jediyi et al. showed activity against *A. niger* of extracts obtained from leaves from different cultivars of *V. vinifera*. Maximum fungal inhibition of 81.81% was observed with the *V. vinifera* superior cultivar. Phenol composition showed high concentration of isoferulic acid 30.98%, quercetine 14.62%, rutine 28.73%, catechin 24.92% and other unidentified phenols [65]. The pure molecule, resveratrol, has been tested against the *Aspergillus* species by different authors. Manimaran et al. showed an inhibition zone of 2 mm for *Aspergillus fumigatus* and of 1.6 mm for *A. niger* at resveratrol concentration of 30 μg/mL [66]. Filip et al. showed antifungal activity of resveratrol at concentration of 11 μg/mL against *A. niger* with a 36.4% growth inhibition [67].

## 5. Antifungal Activity of Crude Extracts, Phenolic and Polyphenolic Compounds from *V. vinifera* Unripe Grapes, Berry Skins, Seeds and Not-Fermented and Fermented Pomaces Against Human Pathogenic Fungi

In *V. vinifera,* the berry and its byproducts, particularly grape seeds, represent the richest matrices of phenols and polyphenols. Moreover, large quantities of wastes from the winery industry, such as unripe grapes and grape pomaces are exploited by some industries to recover bioactive compounds as ingredients for cosmetic formulations, food supplements and medical devices. Antifungal activity of crude extracts of unripe grapes, berry skins, seeds and not-fermented and fermented pomaces was described (Table 2). Unripe grape extracts obtained from agro-industrial wastes, rich in flavan-3-ols (from 3.3 to 6.8 mg/g fresh weight), has been recently evaluated against several strains of *Candida* spp. The geometric mean MIC ranged from 53.58 to 214.31 μg/mL [33]. Grape pomace consisting of seeds, skins and tendrils is an important byproduct of winemaking rich in bioactive phenolic compounds. Pomace extract obtained from non-fermented pomace of *V. vinifera* cv. Italia were rich in caffeic acid, catechin/epicatechin, gallic acid, procyanidins, quinic acid and a series of unknown compounds. The crude pomace extract at 50 µg/mL, loaded in PLGA nanoparticles reduced the mature *Candida* biofilm to 37% [33]. Pomace supercritical fluid extracts from Brazilian Merlot grapes exhibited moderate activity against three *Candida* species (*C. albicans*, *C. krusei* and *C. parapsilosis*), with MIC values ranging from 500 to 1000 μg/mL [68]. Regarding the antifungal activity of GSE obtained from wine and table cultivars of *V. vinifera* L., grown in different agronomic conditions, for the first time, Simonetti et al. [16] demonstrated a significant correlation between anti-*Candida* activity and the content of the flavan-3-ols in GSE, with a polymerization degree ≥ 4. A significant inhibition of *C. albicans,* in an experimental murine model of vaginal candidiasis, using GSE with a high content of polymeric flavan-3-ols has been demonstrated [16]. GSE is generally recognized as safe (GRAS), approved by Food and Drug Administration (FDA) and also sold as a dietary supplement. Antifungal activity together with a lack of toxicity suggests that GSE could be used for the prevention and control of infection diseases without side effects, making greater potential for grapes in the field of food and pharmaceutical application. Cheng et al. showed anti-*Candida* activity of GSE obtained from other varieties of grapes such as Pinot noir and Pinot Meunier showing MIC values of 0.39 and 50 mg/mL, respectively [39]. Yigit and collaborators [69] reported the antifungal activities of the methanol and water extracts of leaf, fruit and seed of *V. vinifera* L. cv. Karaerik against 90 *Candida* strains (*C. albicans*, *C. glabrata* (*Torulopsis glabrata*), *C. guillermondii*, *C. kefyr*, *C. krusei*, *C. parapsilosis*, *C. pseudotropicalis* (*C. kefyr*), *C. tropicalis* and *Geotrichum candidum*). Water extracts of leaves showed the highest anticandidal activity against three *Candida* spp. (*C. albicans*, *C. glabrata* and *C. tropicalis*) with a 20 mm inhibition zone at 1.25 mg/mL MIC [69]. *V. vinifera* seed ethanol extract showed an MIC value of 1000 μg/mL against *C. albicans* and of 500 μg/mL against *C. tropicalis* [70]. Va and collaborators reported that seed ethanolic extract showed activity against *C. albicans* (16 ± 0.8 mm) [51]. Recently, Eslami et al. reported the activity GSE against *C. glabrata* and *C. krusei*; the results showed the same antifungal susceptibility with MIC value of 50 μg/mL [50]. Seed and skin extracts obtained from grape winery byproducts of the Portuguese Arinto variety showed antifungal activity against *C. albicans* [71]. An interesting synergic effect of GSE combined with Amp B against *C. albicans* yeast cells has been demonstrated. Combination of GSE plus Amp B strongly retarded the yeast growth as determined by the broth susceptibility method [72]. Moreover, in the same study it has been shown that GSE enhanced the resistance of mice against the disseminated candidiasis due to *C. albicans* and the polyphenolic compound epigallocatechin gallate contained in GSE caused inhibition of *C. albicans* yeast cell growth. Esposito et al. demonstrated antifungal activity of GSE added to pea protein, in an experimental murine model of *C. albicans* vaginitis, a significantly reduced fungal burden in vagina [73]. Simonetti and collaborators demonstrated the anti-dermatophytes activities of *V. vinifera* seed extracts obtained from several table and wine cultivars. Geometric minimal (GM) inhibitory concentration ranged from 20 to 97 µg/mL for dermatophytes. Dried grape seed extracts analyzed by HPLC/DAD/ESI/MS showed different quali-quantitative compositions in terms of monomeric and polymeric flavan-3-ols. The MIC for *T. mentagrophytes* was inversely correlated with the amount of the polymeric fraction (r = −0.7639). Differently, the antifungal activity against *T. mentagrophytes* was not correlated to the content of flavan-3-ol monomers (r = 0.2920) [20]. Extracts of unripe grapes have been tested against *T. mentagrophytes, T. rubrum* and *M. gypseum*. All extracts showed antifungal activity with GM MIC 80 values ranged from 43.54 to 133.02 μg/mL. Flavan-3-ols were the main metabolites within all samples ranged from 3.3 to 6.8 mg/g fresh weight. For dermatophytes, the highest negative significant correlation has been found between MICs and caffeoyl derivatives (*r* = −0.962; *p* < 0.01). Shrivastav et al. reported the activity of grape extract at 5 μg/μL against *T. rubrum* with an inhibition zone of 7 mm [74].

The yeasts of the genus *Malassezia*, part of the normal microflora, have been associated with several diseases affecting the human skin, such as pityriasis versicolor, folliculitis, dandruff, atopic dermatitis, seborrheic dermatitis, psoriasis and also onychomycosis [75,76]. To date, few therapeutic solutions are available for the treatment of diseases associated with *Malassezia* spp. [77]. Very few studies have been published about the activity of plant extracts against *Malassezia*. Pintas and Quave demonstrated that extracts from several plant species exhibited growth inhibitory activity against the *Malassezia* spp. [78]. About anti-*Malassezia* activity of extracts from *V. vinifera* seeds, only one study is reported in the literature, carried out by Simonetti and colleagues, in which is shown the activity of extracts obtained from different table and wine cultivars. Geometric minimal inhibitory concentration ranged from 32 to 161 µg/mL for *M. furfur*. Dried GSEs analyzed by HPLC/DAD/ESI/MS showed different quali–quantitative compositions in terms of monomeric and polymeric flavan-3-ols. The minimal inhibitory concentration for *M. furfur* was inversely correlated with the amount of the polymeric fraction (r = −0.7228, respectively) and was weakly inversely correlated to the content of flavan-3-ol monomers (r  =  −0.53604) [20].

**Figure 1:**
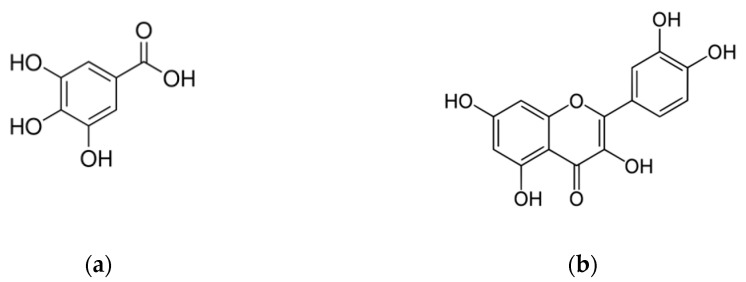

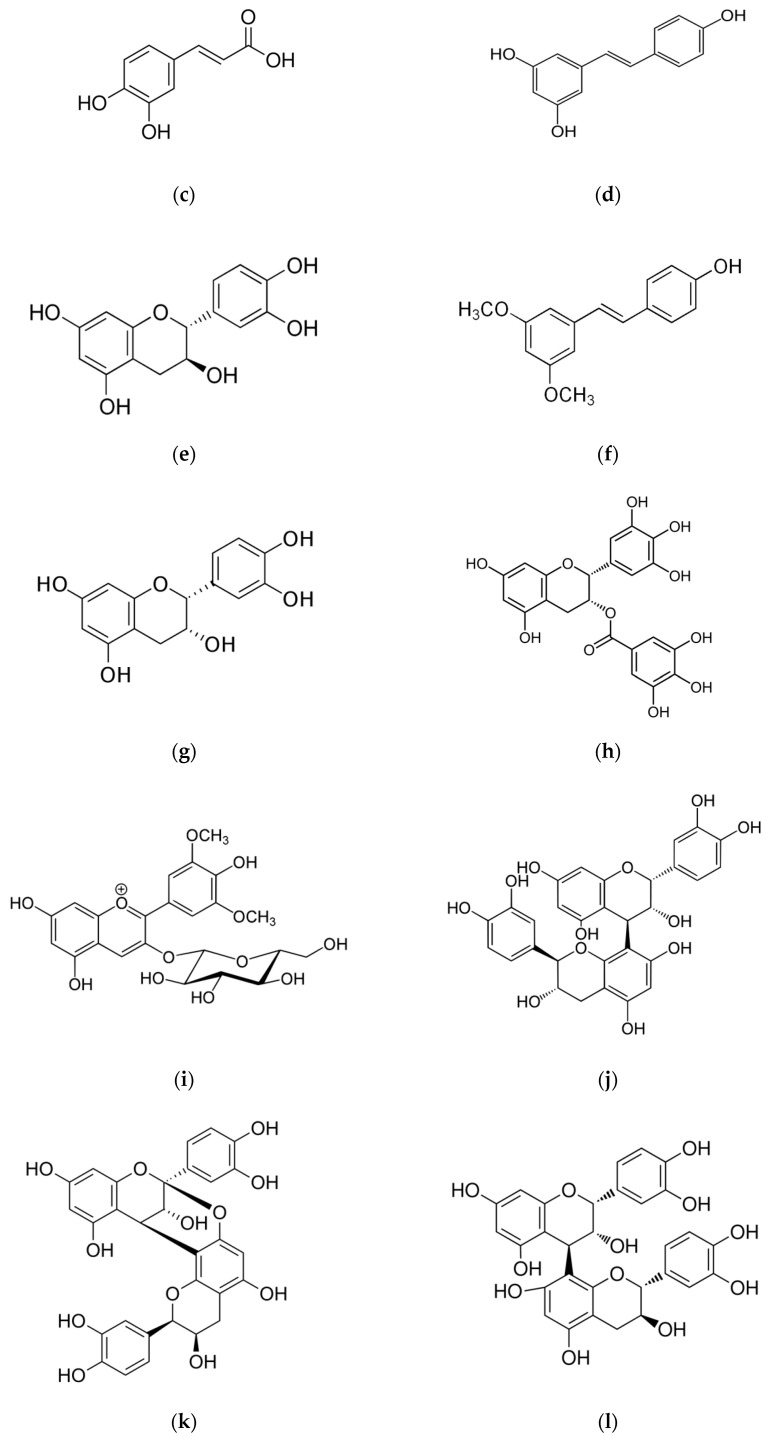
Structures of major phenolic and polyphenolic compounds with antifungal activity identified in different matrices of *V. vinifera,* (**a**) gallic acid, (**b**) quercetin, (**c**) caffeic acid, (**d**), resveratrol, (**e**) catechin, (**f**) pterostilbene, (**g**) epicatechin, (**h**) epigallocatechin-3-gallate, (**i**) malvidin-3-O-glucoside, (**j**) procyanidin B1, (**k**) procyanidin A2 and (**l**) procyanidin B2.

Grape peel extracts obtained using different solvents as water, ethanol, acetone and methanol, have been screened against *A. niger* and *A. versicolor.* The extracts containing higher polyphenolic content (1080 tannic acid equivalent/mL) showed *A. niger* 15% of growth inhibition [79]. In another study, *V. vinifera* grape seed extracts at 1000 μg/mL showed antifungal activity against *Aspergillus* with an inhibition zone of 15.00 ± 0.81 mm [80]. Va et al. also proved that grape seed ethanolic extract had activity against *A. niger* with an inhibition zone of 12 ± 1.6 mm [51]. It should be noted that the *Aspergillus* species are ubiquitous saprophytic fungi to which people are constantly exposed to. Worldwide, aspergillosis affects more than 14 million people. The main diseases are allergic bronchopulmonary aspergillosis (>4 million), severe asthma (>6.5 million), chronic pulmonary aspergillosis (3 million) and invasive aspergillosis (>300,000). Other common diseases are bronchitis, rhinosinusitis, otitis externa and onychomycosis [81]. There are few antifungals in development against *Aspergillus.* The study of plant extract activities represents one possible research to the discovery and development of novel antifungal therapies against *Aspergillus* [82]. *Cryptococcus neoformans* is an opportunistic pathogen that particularly affects people with HIV. There are 223,100 cryptococcal meningitis per year in HIV patients with 181,100 deaths [80]. In the literature there are very few studies on the activity of grape extracts and phenolic compounds. High antifungal activity of GSE, rich in polymeric flavan-3-ols, against a broad panel of human fungal pathogens including *C. neoformans* has been described in a patent of which we are the inventors [83]. In particular, the GSE extract from cultivar Michele Palieri showed a MIC value of 4 µg/mL and total inhibition of *C. neoformans* growth at the concentration of 125.0 µg/mL. Kumar and Vijayalakshmi showed antifungal activity against *C. neoformans* of *V. vinifera* GSE ethanol extract with an MIC value of 1000 μg/mL [70]. In Figure 1 and Table 3 are reported the structures and molecular weight, respectively, of major phenolic and polyphenolic compounds with antifungal activity identified in different matrices of *V. vinifera.*

## 6. Antifungal Mechanisms of Phenolic and Polyphenolic Compounds from *V. vinifera*

Phenolic compounds from *V. vinifera* possess a strong binding ability to different molecular structures like proteins or glycoproteins. Antifungal mechanisms of single phenolic compounds are described (Table 4).

Some authors reported the mechanism of the antifungal activity of stilbenes, such as pterostilbene and resveratrol. Pterostilbene activity was related to the downregulation of the Ras/cAMP pathway and the ergosterol biosynthesis in *C. albicans* [84]. Recently it was demonstrated that resveratrol is an apoptosis inducer in the human pathogenic fungus *C. albicans* activating metacaspase and promoting cytochrome *c* release [85]. Several authors reported the antimicrobial activity of epigallocathechin-3-gallate (EGCG). Navarro-Martinez et al. reported the antifungal activity of EGCG against *C. albicans* ATCC 10231 with an MIC value of 4 µg/mL. Regarding the antifungal mechanism, the authors demonstrated the antifolate activity of EGCG. *C. albicans* dihydrofolate reductase incubated with different concentrations of EGCG showed an inhibition constant (K_i_) of 0.7 µM [86]. Moreover, the authors reported a reduction of ergosterol biosynthesis correlated to the inhibition of folate pathways [86]. The inhibition of ergosterol biosynthesis is also the target of both gallic acid and quercitrin. In particular, gallic acid reduced the activity of sterol 14α-demethylase P450 (CYP51) and squalene epoxidase in *C. albicans,* [87], whereas the quercetin reduced ERG6 expression in *T. rubrum* [88]. Trans-chalcone and quercetin were able to downregulate fatty acid synthase gene expression and reduce ergosterol content in the human pathogenic dermatophyte *T. rubrum* [88]. Moreover, quercitrin in *T. rubrum* inhibited enzymatic activity of fatty acid synthase (FAS) and expression of FAS1 involved in fatty acid biosynthesis.

Cheah and collaborators reported that caffeic acid inhibited isocitrate lyase enzyme that is one of the key enzymes of the glyoxylate cycle and it is an important virulence factor for *C. albicans* [89]. Feldman and colleagues showed that proanthocyanidins were able to reduce the adherence properties of *C. albicans* attenuating the inflammatory response, interfering with NF-κB p65 activation and the phosphorylation of specific signal intracellular kinases [90].

## 7. Applications

The utilization of phenolic and polyphenolic compounds or crude extracts from grape wastes and the byproducts, from the agricultural and agro-industrial process, as a source of functional ingredients to use in formulations against several fungal pathogens, is a promising field. The observed antifungal activity of grape products made them attractive for commercial application. For example, GSE as an active ingredient in the serum form. To enhance absorption and penetration of active ingredients is using the phytosome system [91]. Moreover, GSE, as a source of polyphenols with antioxidant and anti-inflammatory properties, has been proposed to develop formulations both for an anti-aging capability and as a protective capacity against UV and visible light radiation. In this contest GSE and VINEATROL^®^, a commercial extract, containing high quantity of stilbenes, mainly trans resveratrol and trans ε viniferin has been shown to be efficacious for preventing oxidative damages induced by ionizing radiation exposition in diagnostic or radiotherapy purposes [25]. Extracts obtained from waste matrices of the vinicultural chain such as seeds and/or pomace, fermented and not, characterized by a high content of proanthocyanidins compared to the dry weight of the matrix, are particularly active against fungi causing mucocutaneous infections. The results of our studies have led to a Patent IT1407378B1 [83] (Co-Ownership Sapienza University of Rome 80%, Council for Research and Experimentation in Agriculture 20%) relates to the use as antifungal agents of *V. vinifera* extracts. The development of topical formulations containing pomace and seed extracts with antifungal activity is in the process of registration in human and veterinary fields. It is important to point out that these extracts are non-toxic. The main industrial interest is to obtain a phytocomplex with a standardized and reproducible content in characterized active principles, starting from low cost and safe plant matrices and by using simple and reproducible extraction procedures.

## 8. Discussion and Conclusions

The research of plant extracts with biological activity has become an important field of research. The possibility to use plant extracts for human health is a great opportunity but depends on the seriousness that is placed in studying them. Recent findings show antifungal activity of *V. vinifera* extracts, rich in phenols and polyphenols, against human fungal pathogens. We thought to summarize the antifungal activity of *V. vinifera* crude extracts, as a complex mixture of active and inactive natural products, and single molecules. The differences in the results of activity of the extracts demonstrate that it is necessary an approach including a careful design, meticulous execution but above all the choice of the plant matrix, solvent and extraction method. In our experience the content of the extract, in terms of active molecules, depends also on climatic and geographical factors, stages of organ ripeness and cultural practices. For example, chlorogenic acid production, considering its importance in the biosynthetic pathway of polyphenols, in *V. vinifera* plants, varied under ozone exposition, depending on the cultivar. Maturano cv. resulted in being more sensitive to O_3_ with the respect to S. Giuseppe cv. Ozone induced an early chlorogenic acid decrease, which was significantly more consistent in cultivar Maturano [92]. It is important to stress that the origin of plant matrices, the agronomic conditions, a careful description of the extraction process and chemical analysis should always be described. Unfortunately, in many published studies it is not so. For compounds/extracts already used in humans, a dose–response curve should be planned [93]. Moreover, evaluation of antimicrobial activity should be performed using dilution standard tests as reported Clinical and Laboratory Standards Institute (CLSI) guidelines. Diffusion tests are not suitable and not recommended. Triplicates are the lowest number of data for statistics and a suitable statistic should be applied [94]. Based on ours and on some of the reported studies we can conclude that *V. vinifera* extracts, rich in phenols and polyphenols, could be useful to develop novel antifungals.

## Figures and Tables

**Table 1:** Extraction methods, solvent and yield of main procyanidins and total flavan-3-ols obtained from different *V. vinifera* matrices.

Flavan-3-ols	Matrices and Cultivars	Extraction	Solvent	Detection	Range	Ref.
**Procyanidin A2**	Pomace; cultivar Pinot noir	ME	Methanol/water 50:50 (*v*/*v*)	LC–MS	7.80 mg/g of extract	[39]
	Pomace; cultivar Pinot Meunir	ME	Methanol/water 50:50 (*v*/*v*)	LC–MS	11.37 mg/g of extract	[39]
**Procyanidin B1**	Skin; cultivar Michele Palieri	ME	Methanol/water/formic acid 70:30:1 (*v*/*v*/*v*)	LC–MS	184.5–247.8 mg/kg WW	[24]
	Skin; cultivar Red Globe	ME	Methanol/water/formic acid 70:30:1 (*v*/*v*/*v*)	LC–MS	133.5–160.7 mg/kg WW	[24]
	Skin; cultivar Aglianico	ME	Acetone/water 80:20 (*v*/*v*) Methanol/water 60:40 (*v*/*v*)	HPLC/ESI/MS	0.035 mg/g DW	[40]
	Skin; cultivar Merlot	ME	Acetone/water 80:20 (*v*/*v*) Methanol/water 60:40 (*v*/*v*)	HPLC/ESI/MS	0.034 mg/g DW	[40]
	Skin; cultivar Cabernet Sauvignon	ME	Acetone/water 80:20 (*v*/*v*) Methanol/water 60:40 (*v*/*v*)	HPLC/ESI/MS	0.038 mg/g DW	[40]
	Seed; cultivar Michele Palieri	ME	Methanol/water/formic acid 70:30:1 (*v*/*v*/*v*)	LC–MS	1263.1–1643.8 mg/kg WW	[24]
	Seed: cultivar Red Globe	ME	Methanol/water/formic acid 70:30:1 (*v*/*v*/*v*)	LC–MS	414.8–905.2 mg/kg WW	[24]
	Seed; cultivar Aglianico	ME	Acetone/water 80:20 (*v*/*v*) Methanol/water 60:40 (*v*/*v*)	HPLC/ESI/MS	0.065 mg/g DW	[40]
	Seed; cultivar Merlot	ME	Acetone/water 80:20 (*v*/*v*) Methanol/water (60:40, *v*/*v*)	HPLC/ESI/MS	0.059 mg/g DW	[40]
	Seed; cultivar Cabernet Sauvignon	ME	Acetone/water 80:20 (*v*/*v*) Methanol/water 60:40 (*v*/*v*)	HPLC/ESI/MS	0.061 mg/g DW	[40]
	Stem; cultivar Syrah	ASE	Acetone/water 80:20 (*v*/*v*) Methanol/water 60:40 (*v*/*v*)	HPLC-UV-fluo	132.0 mg/100 g DW	[41]
	Stem; cultivar Tempranillo	ASE	Acetone/water 80:20 (*v*/*v*) Methanol/water 60:40 (*v*/*v*)	HPLC-UV-fluo	195.8 mg/100 g DW	[41]
	Stem; cultivar Parellada	ASE	Acetone/water 80:20 (*v*/*v*) Methanol/water 60:40 (*v*/*v*)	HPLC-UV-fluo	187.7 mg/100 g DW	[41]
	Stem; cultivar Premsal Blanc	ASE	Acetone/water 80:20 (*v*/*v*) Methanol/water 60:40 (*v*/*v*)	HPLC-UV-fluo	121.8 mg/100 g DW	[41]
**Procyanidin B2**	Skin; cultivar Michele Palieri	ME	Methanol/water/formic acid 70:30:1 (*v*/*v*/*v*)	LC–MS	17.1–27.4 mg/kg WW	[24]
	Skin; cultivar Red Globe	ME	Methanol/water/formic acid 70:30:1 (*v*/*v*/*v*)	LC–MS	20.2–27.7 mg/kg WW	[24]
	Skin; cultivar Aglianico	ME	Acetone/water 80:20 (*v*/*v*) Methanol/water 60:40 (*v*/*v*)	HPLC/ESI/MS	0.036 mg/g DW	[40]
	Skin; cultivar Merlot	ME	Acetone/water 80:20 (*v*/*v*) Methanol/water 60:40 (*v*/*v*)	HPLC/ESI/MS	0.045 mg/g DW	[40]
	Skin; cultivar Cabernet Sauvignon	ME	Acetone/water 80:20 (*v*/*v*) Methanol/water 60:40 (*v*/*v*)	HPLC/ESI/MS	0.043 mg/g DW	[40]
	Seed; cultivar Michele Palieri	ME	Methanol/water/formic acid 70:30:1 (*v*/*v*/*v*)	LC–MS	1203.6–1636.4 mg/kg WW	[24]
	Seed; cultivar Red Globe	ME	Methanol/water/formic acid 70:30:1 (*v*/*v*/*v*)	LC–MS	394.0–883.1 mg/kg WW	[24]
	Seed; cultivar Aglianico	ME	Acetone/water 80:20 (*v*/*v*) Methanol/water 60:40 (*v*/*v*)	HPLC/ESI/MS	0.078 mg/g DW	[40]
	Seed; cultivar Merlot	ME	Acetone/water 80:20 (*v*/*v*) Methanol/water 60:40 (*v*/*v*)	HPLC/ESI/MS	0.085 mg/g DW	[40]
	Seed; cultivar Cabernet Sauvignon	ME	Acetone/water 80:20 (*v*/*v*) Methanol/water 60:40 (*v*/*v*)	HPLC/ESI/MS	0.083 mg/g DW	[40]
	Stem; cultivar Tempranillo	ASE	Acetone/water 80:20 (*v*/*v*) Methanol/water 60:40 (*v*/*v*)	HPLC-UV-fluo	9.4 mg/100 g DW	[41]
	Stem; cultivar Premsal Blanc	ASE	Acetone/water 80:20 (*v*/*v*) Methanol/water 60:40 (*v*/*v*)	HPLC-UV-fluo	4.0 mg/100 g DW	[41]
**Total** **flavan-3-ols**	Pomace; cultivar Labrusca	ASE	Ethanol/water 50:50 (*v*/*v*)	HPLC-ESI-MS/MS	3253–5708 mg/100 g DW	[36]
	Stem; cultivar Syrah	ASE	Acetone/water 80:20 (*v*/*v*) Methanol/water 60:40 (*v*/*v*)	HPLC-UV-fluo	269.7 mg/100 g DW	[41]
	Stem; cultivar Tempranillo	ASE	Acetone/water 80:20 (*v*/*v*) Methanol/water 60:40 (*v*/*v*)	HPLC-UV-fluo	366.3 mg/100 g DW	[41]
	Stem; cultivar Premsal Blanc	ASE	Acetone/water 80:20 (*v*/*v*) Methanol/water 60:40 (*v*/*v*)	HPLC-UV-fluo	214.2 mg/100 g DW	[41]
	Seed; cultivar Michele Palieri	ME	Ethanol/water/formic acid 70:30:1 (*v/v*/*v*)	HPLC/ESI/MS	638.5 mg/g of extract	[16]
	Unripe grape; cultivar Don Mariano	ME	Ethanol/water/formic acid 70:30:1 (*v*/*v*/*v*)	HPLC/ESI/MS	2666 µg/g FW	[33]
	Unripe grape; cultivar Alphonse Lavalle	ME	Ethanol/water/formic acid 70:30:1 (*v*/*v*/*v*)	HPLC/ESI/MS	6773 µg/g FW	[33]
	Unripe grape; cultivar Italia	ME	Ethanol/water/formic acid 70:30:1 (*v*/*v*/*v*)	HPLC/ESI/MS	3390 µg/g FW	[33]
	Unripe grape; cultivar Michele Palieri	ME	Ethanol/water/formic acid 70:30:1 (*v*/*v*/*v*)	HPLC/ESI/MS	3596 µg/g FW	[33]
	Unripe grape; cultivar Red Globe	ME	Ethanol/water/formic acid 70:30:1 (*v*/*v*/*v*)	HPLC/ESI/MS	2300 µg/g FW	[33]
	Unripe grape; cultivar Almeria	ME	Ethanol/water/formic acid 70:30:1 (*v*/*v*/*v)*	HPLC/ESI/MS	4149 µg/g FW	[33]

ME: extraction by maceration, ASE: accelerated solvent extraction; WW: wet weight; DW: dried weight; FW: fresh weight.

**Table 2:** Antifungal activities of crude extracts, phenolic and polyphenolic compounds obtained from different matrices of *V. vinifera* against human fungal pathogens. spp. = species; MIC = Minimal Inhibitory Concentration (µg/mL); CFU = Colony Forming Unit. Nd: not declared.

	Matrices	Extraction	Detection	Compounds	Microorganism	MIC µg/mL	Ref.
**Seeds**	Cultivar Michele Palieri Turi (BA) Italy, 2010 treated with water 2000 m^3^/hectare and nitrogen fertilization 120 kg/hectare	Ethanol/water 70:30 (*v*/*v*)	HPLC/ESI/MS	Flavan-3-ols 820 mg/g	*Candida albicans* ATCC 10231	8	[16]
			HPLC/ESI/MS		*Candida krusei* DSM 6128	4	[16]
			HPLC/ESI/MS		*Candida parapsilosis* ATCC 22019	8	[16]
			HPLC/ESI/MS		*Candida glabrata* PMC 0805	4	[16]
			HPLC/ESI/MS		*Candida tropicalis* DSM 11953	16	[16]
			HPLC/DAD/ESI/MS		*Trichophyton mentagrophytes* DSM 4870	32	[20]
			HPLC/DAD/ESI/MS		*Microsporum gypseum* PMC 7331	64	[20]
			HPLC/DAD/ESI/MS		*Microsporum canis* PMC 7426	64	[20]
	Cultivar Michele Palieri Turi (BA) Italy, 2012	Ethanol/water	HPLC/DAD/ESI/MS	Flavan-3-ols 383.4 mg/g	*Malassezia furfur* DSM 6171	16	[20]
		Ethanol	HPLC/DAD/ESI/MS	Flavan-3-ols 272.0 mg/g	*Malassezia furfur* DSM 6171	64	[20]
		Methanol	HPLC/DAD/ESI/MS	Flavan-3-ols 288.2 mg/g	*Malassezia furfur* DSM 6171	32	[20]
	Nd	Ethanol/water 70:30 (*v*/*v*)	Nd	Nd	*Candida glabrata* BSM 11226	50	[50]
	Nd	Ethanol/water 70:30 (*v*/*v*)	Nd	Nd	*Candida krusei* BSM 70079	50	[50]
	Wine residue obtained after 1 week of pre-fermentation from Pinot noir, Lincoln University, Canterbury, New Zealand, between 2008 and 2009	Ethanol/water 50:50 (*v*/*v*)	LC–MS	Catechin 14.31 mg/g	*Candida albicans* ATCC 10261	390	[39]
		Acetone/water 50:50 (*v*/*v*)	LC–MS	Catechin 13.41mg/g	*Candida albicans* ATCC 10261	780	[39]
		Methanol/water 50:50 (*v*/*v*)	LC–MS	Catechin 15.18 mg/g	*Candida albicans* ATCC 10261	780	[39]
	Nd	Ethanol	Nd	Nd	*Candida albicans* MTCC 227	1000	[70]
	Nd	Ethanol	Nd	Nd	*Candida tropicalis* MTCC 184	500	[70]
	Nd	Ethanol	Nd	Nd	*Cryptococcus neoformans* clinical isolate	1000	[70]
**Pomace**	Wine residue obtained after 1 week of pre-fermentation from Pinot noir, Lincoln University, Canterbury, New Zealand, between 2008 and 2009	Ethanol/water 50:50 (*v*/*v*)	LC–MS	Catechin 8.97 mg/g	*Candida albicans* ATCC 10261	390	[39]
		Acetone/water 50:50 (*v*/*v*)	LC–MS	Catechin 10.01 mg/g	*Candida albicans* ATCC 10261	>1000	[39]
		Methanol/water 50:50 (*v*/*v*)	LC–MS	Catechin 11.81 mg/g	*Candida albicans* ATCC 10261	>1000	[39]
	Merlot pomace, vintage 2009, Vale dos Vinhedos–Rio Grande do Sul, Brazil	Supercritical fluid extraction SFE SC-CO_2_ 50 °C/200 bar	RF-HPLC-UV	Gallic acid 1159	*Candida albicans* ATCC 14053	500	[49]
			RF-HPLC-UV		*Candida parapsilosis* ATCC 22019	1000	[49]
			RF-HPLC-UV		*Candida krusei* ATCC 6258	500	[49]
	non-fermented pomace cultivar Italia grown in the experimental farm of Crea-Utv, Turi (Bari) Italy	Ethanol/water 70:30 (*v*/*v*)	HPLC/ESI/MS	Procyanidins 90.67 mg/g	*Candida albicans* ATCC 10231	12.5	[54]
			HPLC/ESI/MS		*Candida albicans* 3135	6.25	[54]
			HPLC/ESI/MS		*Candida albicans* ATCC 20891	1.4	[54]
			HPLC/ESI/MS		*Candida albicans* ATCC 10261	12.5	[54]
**Skin**	Wine residue obtained after 1 week of pre-fermentation from Pinot noir, Lincoln University, Canterbury, New Zealand, between 2008 and 2009	Ethanol/water 50:50 (*v*/*v*)	LC–MS	Malvidin-3-glucoside 8.36 mg/g	*Candida albicans* ATCC 10261	780	[39]
		Acetone/water 50:50 (*v*/*v*)	LC–MS	Malvidin-3-glucoside 10.64 mg/g	*Candida albicans* ATCC 10261	780	[39]
		Methanol/water 50:50 (*v*/*v*)	LC–MS	Malvidin-3-glucoside 9.95 mg/g	*Candida albicans* ATCC10261	780	[39]
**Unripe** **grape**	Alphonse Lavalle experimental farm of Crea-Utv, Turi (Bari) Italy	Ethanol/water 70:30 (*v*/*v*)	HPLC/ESI/MS	Polymeric flavan-3-ols 3896 μg/g FW	*Candida albicans* ATCC 10231	64	[33]
					*Candida krusei* DSM 6128	64	[33]
					*Candida glabrata* PMC 0805	64	[33]
					*Candida parapsilosis* ATCC 22019	64	[33]
					*Candida tropicalis* DSM 11953	128	[33]
					*Microsporum gypseum* PMC 7331	16	[33]
					*Triciphyton mentagrophytes* PMC 6503	64	[33]
					*Tricophyton rubrum* PMC 6612	64	[33]
**Leaves**	2014 from Zabol region (Coordinates: 31°1′43″ N, 61°30′4″ E), in Sistan and Baluchestan Provinces of Iran.	Methanol	Nd	Nd	*Candida albicans* ATCC 10231	80.9	[64]
			Nd	Nd	*Aspergillus niger* ATCC 9142	75.4	[64]

**Table 3:** Molecular weight of phenolic and polyphenolic compounds from *V. vinifera* with antifungal activity.

Chemical Compounds	Molecular Weight
Gallic acid	170.1
Caffeic acid	180.2
Catechin	290.3
Epicatechin	290.3
Epicatechin-3-gallate	442.4
Quercetin	448.4
Resveratrol	228.2
Pterostilbene	256.3
Malvidin-3-O-glucoside	493.4
Procyanidin A2	576.5
Procyanidin B1	578.5
Procyanidin B2	578.5

**Table 4:** Antifungal mechanisms of single phenolic compounds against human fungal pathogens.

Compounds	*Fungi*	Antifungal Mechanisms	Ref.
Pterostilbene	*Candida albicans*	Down regulator of Ras/cAMP pathway and the ergosterol biosynthesis.	[84]
Resveratrol	*Candida albicans*	Activator of metacaspase and cytochrome *c* release inducing apoptosis	[85]
Epigallocatechin-3-gallate	*Candida albicans*	Inhibitor of dihydrofolate reductase (*K*i = 0.7 µM). Synergy with inhibitors of the ergosterol biosynthesis pathway	[86]
Gallic acid	*Trichophyton rubrum*	Inhibitor of ergosterol biosynthesis reducing the activity of sterol 14α-demethylase P450 (CYP51) and squalene epoxidase	[87]
Quercetin	*Trichophyton rubrum*	Reduces ergosterol levels and modulates the expression of *FAS1* and *ERG6*	[88]
Caffeic acid	*Candida albicans*	Inhibits isocitrate lyase enzyme activity	[89]
Proanthocyanidins	*Candida albicans*	Reduce the adherence properties of fungus attenuating the inflammatory response, interfering with NF-κB p65 activation and the phosphorylation of specific signal intracellular kinases.	[90]

## References

[1] Gintjee T.J., Donnelley M.A., Thompson G.R. (2020). Aspiring Antifungals: Review of Current Antifungal Pipeline Developments. J. Fungi.

[2] Carvalho R.S., Carollo C.A., de Magalhães J.C., Palumbo J.M.C., Boaretto A.G., Sá I.N., Ferraz A.C., Limaa W.G., de Siqueira J.M., Ferreira J.M.S. (2018). Antibacterial and antifungal activities of phenolic compound-enriched ethyl acetate fraction from *Cochlospermum regium* (mart. Et. Schr.) Pilger roots: Mechanisms of action and synergism with tannin and gallic acid. S. Afr. J. Bot..

[3] Brown G.D., Denning D.W., Levitz S.M. (2012). Tackling human fungal infections. Science.

[4] Mandras N., Nostro A., Roana J., Scalas D., Banche G., Ghisetti V., Del Re S., Fucale G., Cuffini A.M., Tullio V. (2016). Liquid and vapour-phase antifungal activities of essential oils against *Candida albicans* and non-albicans *Candida*. BMC Complem. Altern..

[5] Tullio V., Nostro A., Mandras N., Dugo P., Banche G., Cannatelli M.A., Cuffini A.M., Alonzo V., Carlone N.A. (2007). Antifungal activity of essential oils against filamentous fungi determined by broth microdilution and vapour contact methods. J. Appl. Microbiol..

[6] Redondo-Blanco S., Fernández J., López-Ibáñez S., Miguélez E., Villar C., Lombò F. (2020). Plant phytochemicals in food preservation: Antifungal bioactivity: A review. J. Food Prot..

[7] Zida A., Bamba S., Yacouba A., Ouedraogo-Traore R., Guiguemdé R.T. (2017). Anti-*Candida albicans* natural products, sources of new antifungal drugs: A review. J. Mycol. Med..

[8] Friedman M. (2014). Antibacterial, antiviral, and antifungal properties of wines and winery byproducts in relation to their flavonoid content. J. Agric. Food Chem..

[9] Fraternale D., Ricci D., Verardo G., Gorassini A., Stocchi V., Sestili P. (2015). Activity of *Vitis vinifera* tendrils extract against phytopathogenic fungi. Nat. Prod. Commun..

[10] Olas B. (2018). Berry phenolic antioxidants–implications for human health?. Front Pharmacol..

[11] Durazzo A., Lucarini M., Souto E.B., Cicala C., Caiazzo E., Izzo A.A., Novellino E., Santini A. (2019). Polyphenols: A concise overview on the chemistry, occurrence, and human health. Phytother. Res..

[12] Veiga M., Costa E.M., Silva S., Pintado M. (2020). Impact of plant extracts upon human health: A review. Crit. Rev. Food Sci. Nutr..

[13] Panzella L., Moccia F., Nasti R., Marzorati S., Verotta L., Napolitano A. (2020). Bioactive phenolic compounds from agri-food wastes: An update on green and sustainable extraction methodologies. Front. Nutr..

[14] Garrido J., Borges F. (2013). Wine and grape polyphenols. A chemical perspective. Food Res. Int..

[15] Haselgrove L., Botting D., Van Heeswijck R., Høj P.B., Dry P.R., Ford C., Land P.G.I. (2000). Canopy microclimate and berry composition: The effect of bunch exposure on the phenolic composition of *Vitis vinifera* L cv. Shiraz grape berries. Aust. J. Grape Wine Res..

[16] Simonetti G., Santamaria A.R., D’Auria F.D., Mulinacci N., Innocenti M., Cecchini F., Pericolini E., Gabrielli E., Panella S., Antonacci D. (2014). Evaluation of anti-*Candida* activity of *Vitis vinifera* L. seed extracts obtained from wine and table cultivars. BioMed Res. Int..

[17] Giannini B., Mulinacci N., Pasqua G., Innocenti M., Valletta A., Cecchini F. (2016). Phenolics and antioxidant activity in different cultivars/clones of *Vitis vinifera* L. seeds over two years. Plant Biosyst..

[18] Cheynier V., Rigaud J. (1986). HPLC separation and characterization of flavonols in the skins of *Vitis vinifera* var. Cinsault. Am. J. Enol. Vitic..

[19] Souquet J.M., Labarbe B., Le Guernevé C., Cheynier V., Moutounet M. (2000). Phenolic composition of grape stems. J. Agric. Food Chem..

[20] Simonetti G., D’Auria F.D., Mulinacci N., Innocenti M., Antonacci D., Angiolella L., Pasqua G. (2017). Anti-Dermatophyte and Anti-Malassezia Activity of Extracts Rich in Polymeric Flavan-3-ols Obtained from *Vitis vinifera* Seeds. Phytother. Res..

[21] Fontana A.R., Antoniolli A., Bottini R. (2013). Grape pomace as a sustainable source of bioactive compounds: Extraction, characterization, and biotechnological applications of phenolics. J. Agric. Food Chem..

[22] Ghafoor K., AL-Juhaimi F.Y., Choi Y.H. (2012). Supercritical fluid extraction of phenolic compounds and antioxidants from grape (*Vitis labrusca* B.) seeds. Plant Foods Hum. Nutr..

[23] Fuleki T., Ricardo da Silva J.M. (1997). Catechin and procyanidin composition of seeds from grape cultivars grown in Ontario. J. Agric. Food Chem..

[24] Cavaliere C., Foglia P., Marini F., Samperi R., Antonacci D., Laganà A. (2010). The interactive effects of irrigation, nitrogen fertilisation rate, delayed harvest and storage on the polyphenol content in red grape (*Vitis vinifera*) berries: A factorial experimental design. Food Chem..

[25] Mulinacci N., Valletta A., Pasqualetti V., Innocenti M., Giuliani C., Bellumori M., De Angelis G., Carnevale A., Locato V., Di Venanzio C. (2019). Effects of ionizing radiation on bio-active plant extracts useful for preventing oxidative damages. Nat. Prod. Res..

[26] Dinicola S., Cucina A., Pasqualato A., D’Anselmi F., Proietti S., Lisi E., Pasqua G., Antonacci D., Bizzarri M. (2012). Antiproliferative and apoptotic effects triggered by grape seed extract (GSE) versus epigallocatechin and procyanidins on colon cancer cell lines. Int. J. Mol. Sci..

[27] Giovannelli L., Innocenti M., Santamaria A.R., Bigagli E., Pasqua G., Mulinacci N. (2014). Antitumoural activity of viniferin-enriched extracts from *Vitis vinifera* L. cell cultures. Nat. Prod. Res..

[28] Pasqua G., Simonetti G. (2016). Antimicrobial and antiviral activities of grape seed extracts. Grape Seeds.

[29] Goufo P., Singh R.K., Cortez I. (2020). A Reference List of Phenolic Compounds (Including Stilbenes) in Grapevine (*Vitis vinifera* L.) Roots, Woods, Canes, Stems, and Leaves. Antioxidants.

[30] Moldovan M.L., Bogdan C., Lurian S., Roman C., Oniga I., Benedec D. (2020). Phenolic content and antioxidant capacity of pomace and canes extracts of some *Vitis vinifera* varieties cultivated in Romania. Farmacia.

[31] Moldovan M.L., Carpa R., Fizeșan I., Vlase L., Bogdan C., Iurian S.M., Benedec D., Pop A. (2020). Phytochemical Profile and Biological Activities of Tendrils and Leaves Extracts from a Variety of *Vitis vinifera* L.. Antioxidants.

[32] Di Lorenzo R., Gambino C., Scafidi P. (2011). Summer pruning in table grape. Adv. Hortic. Sci..

[33] Simonetti G., D’Auria F.D., Mulinacci N., Milella R.A., Antonacci D., Innocenti M., Pasqua G. (2019). Phenolic content and in vitro antifungal activity of unripe grape extracts from agro-industrial wastes. Nat. Prod. Res..

[34] Lucarini M., Durazzo A., Kiefer J., Santini A., Lombardi-Boccia G., Souto E.B., Romani A., Lampe A., Ferrari Nicoli S., Gabrielli P. (2020). Grape seeds: Chromatographic profile of fatty acids and phenolic compounds and qualitative analysis by FTIR-ATR spectroscopy. Foods.

[35] González-Centeno M.R., Rosselló C., Simal S., Garau M.C., López F., Femenia A. (2010). Physico-chemical properties of cell wall materials obtained from ten grape varieties and their byproducts: Grape pomaces and stems. LWT Food Sci. Technol..

[36] Monrad J.K., Howard L.R., King J.W., Srinivas K., Mauromoustakos A. (2010). Subcritical solvent extraction of procyanidins from dried red grape pomace. J. Agric. Food Chem..

[37] González-Centeno M.R., Knoerzer K., Sabarez H., Simal S., Rosselló C., Femenia A. (2014). Effect of acoustic frequency and power density on the aqueous ultrasonic-assisted extraction of grape pomace (*Vitis vinifera* L.) a response surface approach. Ultrason. Sonochem..

[38] García-Lomillo J., González-San José M.L. (2017). Applications of wine pomace in the food industry: Approaches and functions. Compr. Rev. Food Sci. F.

[39] Cheng V.J., Bekhit A.E.D.A., McConnell M., Mros S., Zhao J. (2012). Effect of extraction solvent, waste fraction and grape variety on the antimicrobial and antioxidant activities of extracts from wine residue from cool climate. Food Chem..

[40] Rinaldi A., Jourdes M., Teissedre P.L., Moio L. (2014). A preliminary characterization of Aglianico (*Vitis vinifera* L. cv.) grape proanthocyanidins and evaluation of their reactivity towards salivary proteins. Food Chem..

[41] González-Centeno M.R., Jourdes M., Femenia A., Simal S., Rosselló C., Teissedre P.L. (2012). Proanthocyanidin composition and antioxidant potential of the stem winemaking byproducts from 10 different grape varieties (*Vitis vinifera* L.). J. Agric. Food Chem..

[42] Yilmaz Y., Toledo R.T. (2006). Oxygen radical absorbance capacities of grape/wine industry byproducts and effect of solvent type on extraction of grape seed polyphenols. J. Food Compos. Anal..

[43] Youssef D., El-Adawi H. (2006). Study on grape seeds extraction and optimization: An approach. J. Appl. Sci..

[44] Vergara-Salinas J.R., Bulnes P., Zúñiga M.C., Pérez-Jiménez J., Torres J.L., Mateos-Martín M.L., Agosin E., Pérez-Correa J.R. (2013). Effect of pressurized hot water extraction on antioxidants from grape pomace before and after enological fermentation. J. Agric. Food Chem..

[45] Nawaz H., Shi J., Mittal G.S., Kakuda Y. (2006). Extraction of polyphenols from grape seeds and concentration by ultrafiltration. Sep. Purif. Technol..

[46] Lafka T.-I., Sinanoglou V., Lazos E.S. (2007). On the extraction and antioxidant activity of phenolic compounds from winery wastes. Food Chem..

[47] Makris D.P., Boskou G., Andrikopoulos N.K. (2007). Recovery of antioxidant phenolics from white vinification solid by-products employing water/ethanol mixtures. Biores. Technol..

[48] Bucić-Kojić A., Planinić M., Tomas S., Bilić M., Velić D. (2007). Study of solid–liquid extraction kinetics of total polyphenols from grape seeds. J. Food Eng..

[49] Oliveira D.A., Salvador A.A., Smânia A., Smânia E.F., Maraschin M., Ferreira S.R. (2013). Antimicrobial activity and composition profile of grape (*Vitis vinifera*) pomace extracts obtained by supercritical fluids. J. Biotechnol..

[50] Eslami H., Babaei H., Mehrbani S.P., Aghazadeh M., Babaei Z., Nezhad S.K. (2017). Evaluation of antifungal effect of grape seed extract (GSE) on *Candida glabrata* and *Candida krusei*: In vitro study. Biomed. Res..

[51] Va M., Eb J.E. (2016). The Journal of Free Radicals and Antioxidants. Photon.

[52] Kolouchová I., Maťátková O., Paldrychová M., Kodeš Z., Kvasničková E., Sigler K., Čejková A., Šmidrkal J., Demnerová K., Masák J. (2018). Resveratrol, pterostilbene, and baicalein: Plant-derived anti-biofilm agents. Folia Microbiol..

[53] Ruhnke M. (2019). Clinical Syndromes: Candida and Candidosis. Clinically Relevant Mycoses.

[54] Simonetti G., Palocci C., Valletta A., Kolesova O., Chronopoulou L., Donati L., Di Nitto A., Brasili E., Tomai P., Gentili A. (2019). Anti-Candida biofilm activity of pterostilbene or crude extract from non-fermented grape pomace entrapped in biopolymeric nanoparticles. Molecules.

[55] Jung H.J., Hwang I.A., Sung W.S., Kang H., Kang B.S., Seu Y.B., Lee D.G. (2005). Fungicidal effect of resveratrol on human infectious fungi. Arch. Pharm. Res..

[56] Chan M.M.Y. (2002). Antimicrobial effect of resveratrol on dermatophytes and bacterial pathogens of the skin. Biochem. Pharmacol..

[57] Paulo L., Oleastro M., Gallardo E., Queiroz J.A., Domingues F. (2011). Antimicrobial properties of resveratrol: A review. Science Against Microbial Pathogens: Communicating Current Research and Technological Advances.

[58] Khurana A., Sardana K., Chowdhary A. (2019). Antifungal resistance in dermatophytes: Recent trends and therapeutic implications. Fungal Genet Biol..

[59] Ventoulis I., Sarmourli T., Amoiridou P., Mantzana P., Exindari M., Gioula G., Vyzantiadis T.A. (2020). Bloodstream Infection by Saccharomyces cerevisiae in Two COVID-19 Patients after Receiving Supplementation of Saccharomyces in the ICU. J. Fungi.

[60] Hay R.J., Moore M.K., Burns T., Breathnach S., Cox N., Griffiths C. (2004). Mycology. Rook’s Textbook of Dermatology.

[61] Houille B., Papon N., Boudesocque L., Bourdeaud E., Besseau S., Courdavault V., Clastre M. (2014). Antifungal activity of resveratrol derivatives against *Candida* species. J. Nat. Prod..

[62] Weber K., Schulz B., Ruhnke M. (2011). Resveratrol and its antifungal activity against *Candida* species. Mycoses.

[63] Barac A., Kosmidis C., Alastruey-Izquierdo A., Salzer H.J. (2019). Chronic pulmonary aspergillosis update: A year in review. Med. Mycol..

[64] Sharifi-Rad J., Miri A., Sharifi-Rad M., Sharifi-Rad M., Hoseini-Alfatemi S.M., Yazdanpanah E. (2014). Antifungal and antibacterial properties of Grapevine (*Vitis vinifera* L.) leaves methanolic extract from Iran-in vitro study. Am. Eurasian J. Agric. Environ. Sci..

[65] Jediyi H., Naamani K., Ait Elkoch A., Dihazi A., Lemjiber N. (2020). A comparative study of phenols composition, antioxidant, and antifungal potency of leaves extract from five Moroccan *Vitis vinifera* L. varieties. J. Food Saf..

[66] Manimaran M., Sivakumari K., Ashok K., Rajesh S. (2017). Evaluation of the in vitro antimicrobial effect of resveratrol on human pathogens. Evaluation.

[67] Filip V., Plockova M., Šmidrkal J., Špičková Z., Melzoch K., Schmidt Š. (2003). Resveratrol and its antioxidant and antimicrobial effectiveness. Food Chem..

[68] Peixoto C.M., Dias M.I., Alves M.J., Calhelha R.C., Barros L., Pinho S.P., Ferreira I.C. (2018). Grape pomace as a source of phenolic compounds and diverse bioactive properties. Food Chem..

[69] Yıgıt D., Yıgıt N., Mavı A., Yıldırım A., Güleryüz M. (2009). Antioxidant and antimicrobial activities of methanol and water extracts of fruits, leaves and seeds of *Vitis vinifera* L. cv. Karaerik. Asian J. Chem..

[70] Kumar K.A., Vijayalakshmi K. (2013). In vitro anti-microbial activity and phytochemical analysis of selected fruit wastes. Int. J. Curr. Microbiol. App. Sci..

[71] Kiraly-Veghely Z., Moricz A.M., Ott P.G., Katay G., Belai I., Tyihák E. (2009). Comparison of components from red and white wines for antimicrobial activity by biodetection after OPLC separation. J. Liq. Chromatogr. R. T.

[72] Han Y. (2007). Synergic effect of grape seed extract with amphotericin B against disseminated candidiasis due to *Candida albicans*. Phytomedicine.

[73] Esposito E., Campolo M., Casili G., Lanza M., Filippone A., Peritore A.F., Cuzzocrea S. (2018). Effect of pea protein plus grape seed dry extract on a murine model of Candida albicans induced vaginitis. Future Microbiol..

[74] Shrivastav V.K., Shukla D., Parashar D., Shrivastav A. (2013). Dermatophytes and related keratinophilic fungi isolated from the soil in Gwalior region of India and in vitro evaluation of antifungal activity of the selected plant extracts against these fungi. J. Med. Plant Res..

[75] Gupta A.K., Batra R., Bluhm R., Boekhout T., Dawson T.L. (2004). Skin diseases associated with *Malassezia* species. J. Am. Acad. Dermatol..

[76] Saunte D.M., Gaitanis G., Hay R.J. (2020). Malassezia-Associated Skin Diseases, the Use of Diagnostics and Treatment. Front. Cell. Infect. Microbiol..

[77] Gupta A.K., Versteeg S.G. (2017). Topical treatment of facial seborrheic dermatitis: A systematic review. Am. J. Clin. Dermatol..

[78] Pintas S.K., Quave C.L. (2019). A Review of Botanicals Exhibiting Antifungal Activity Against *Malassezia* spp. Implicated in Common Skin Conditions. Curr. Dermatol. Rep..

[79] Yadav D., Kumar A., Kumar P., Mishra D. (2015). Antimicrobial properties of black grape (*Vitis vinifera* L.) peel extracts against antibiotic-resistant pathogenic bacteria and toxin producing molds. Indian J. Pharmacol..

[80] Ghouila Z., Laurent S., Boutry S., Vander Elst L., Nateche F., Muller R.N., Baaliouamer A. (2017). Antioxidant, antibacterial and cell toxicity effects of polyphenols *Fromahmeur bouamer* grape seed extracts. J. Fundam. Appl. Sci..

[81] Gago S., Denning D.W., Bowyer P. (2019). Pathophysiological aspects of *Aspergillus* colonization in disease. Med. Mycol..

[82] Marquez L., Quave C.L. (2020). Prevalence and Therapeutic Challenges of Fungal Drug Resistance: Role for Plants in Drug Discovery. Antibiotics.

[83] Pasqua G., Simonetti G., D’Auria F.D., Santamaria A.R., Antonacci D. (2014). Extracts Obtained from Seeds and/or Skins of *Vitis vinifera* and Related Uses as Antifungal Agents. Italy Patent.

[84] Li D.D., Zhao L.X., Mylonakis E., Hu G.H., Zou Y., Huang T.K., Yan L., Wang Y., Jiang Y.Y. (2014). In vitro and in vivo activities of pterostilbene against *Candida albicans* biofilms. Antimicrob. Agents Chemother..

[85] Lee J., Lee D.G. (2015). Novel antifungal mechanism of resveratrol: Apoptosis inducer in *Candida albicans*. Curr. Microbiol..

[86] Navarro-Martínez M.D., García-Cánovas F., Rodriguez-Lopez J.N. (2006). Tea polyphenol epigallocatechin-3-gallate inhibits ergosterol synthesis by disturbing folic acid metabolism in *Candida albicans*. J. Antimicrob. Chemother..

[87] Li Z.J., Liu M., Dawuti G., Dou Q., Ma Y., Liu H.G., Aibai S. (2017). Antifungal activity of gallic acid in vitro and in vivo. Phytother. Res..

[88] Bitencourt T.A., Komoto T.T., Massaroto B.G., Miranda C.E.S., Beleboni R.O., Marins M., Fachin A.L. (2013). Trans-chalcone and quercetin down-regulate fatty acid synthase gene expression and reduce ergosterol content in the human pathogenic dermatophyte *Trichophyton rubrum*. BMC Complement. Altern. Med..

[89] Cheah H.L., Lim V., Sandai D. (2014). Inhibitors of the glyoxylate cycle enzyme ICL1 in *Candida albicans* for potential use as antifungal agents. PLoS ONE.

[90] Feldman M., Tanabe S., Howell A., Grenier D. (2012). Cranberry proanthocyanidins inhibit the adherence properties of *Candida albicans* and cytokine secretion by oral epithelial cells. BMC Complement. Altern. Med..

[91] Surini S., Mubarak H., Ramadon D. (2018). Cosmetic serum containing grape (*Vitis vinifera* L.) seed extract phytosome: Formulation and in vitro penetration study. J. Young Pharm..

[92] Valletta A., Salvatori E., Rita Santamaria A., Nicoletti M., Toniolo C., Caboni E., Pasqua G., Manes F. (2016). Ecophysiological and phytochemical response to ozone of wine grape cultivars of *Vitis vinifera* L.. Nat. Prod. Res..

[93] Nair A.B., Jacob S. (2016). A simple practice guide for dose conversion between animals and human. J. Basic Clin. Pharm..

[94] Heinrich M., Appendino G., Efferth T., Fürst R., Izzo A.A., Kayser O., Pezzuto J.M., Viljoen A. (2020). Best practice in research–overcoming common challenges in phytopharmacological research. J. Ethnopharmacol..

